# Dose Characterization of the Investigational Anticancer Drug Tigilanol Tiglate (EBC-46) in the Local Treatment of Canine Mast Cell Tumors

**DOI:** 10.3389/fvets.2019.00106

**Published:** 2019-04-09

**Authors:** Jane Miller, Justine Campbell, Andrew Blum, Paul Reddell, Victoria Gordon, Peter Schmidt, Stewart Lowden

**Affiliations:** ^1^Newtown Veterinary Clinic, Newtown, VIC, Australia; ^2^Tableland Veterinary Services, Atherton, QLD, Australia; ^3^Greencross Vets Palm Beach, Palm Beach, QLD, Australia; ^4^QBiotics Group Ltd, Yungaburra, QLD, Australia; ^5^QBiotics Group Ltd, Taringa, QLD, Australia

**Keywords:** Tigilanol tiglate, EBC-46, mast cell tumor, protein kinase C, solid tumors, intratumoral injection

## Abstract

Mast cell tumor (MCT) is the most common cutaneous neoplasm in dogs and wide surgical resection is the current first-line treatment. However, recurrence is common and often requires more specialist and expensive therapies. Tigilanol tiglate is a novel small molecule drug delivered by intratumoral injection that is currently under development to provide a new option for treating MCT. The aim of this study was to characterize a safe and effective dose of tigilanol tiglate for canine MCT and to gather preliminary data on the drug's pharmacokinetics. A multicenter, open-label, uncontrolled, non-randomized, dose de-escalation design was used. Eligibility was MCT stage I/IIa and a tumor size of 0.1–6.0 cm^3^. Dosing was based on tumor size (50% v/v tumor) and 3 drug concentrations (1.0, 0.5, 0.2 mg/mL) were evaluated. Twenty-seven dogs were treated in 3 dose de-escalation cohorts (10, 10, and 7 dogs, respectively). Efficacy at 21 days was defined using international accepted solid tumor response criteria (RECIST). Greatest efficacy (90% complete response) was observed at the highest drug concentration (1.0 mg/mL) and adverse events were generally low grade, mild and transient, and directly associated with the mode of action of the drug. Hematological and serum biochemistry were generally unremarkable with plasma concentration curves typical of a non-intravenous parenteral medication. Intratumoral treatment of MCT with tigilanol tiglate at a concentration of 1.0 mg/mL was highly efficacious and well-tolerated. These results support the drug's further development for the treatment of MCT and other solid tumors.

## Introduction

Mast cell tumor (MCT) is the most common cutaneous neoplasm in dogs with an estimated prevalence of 0.25–0.27% (1, 2). MCTs account for 16–21% of all cutaneous neoplasms in dogs (3, 4). Breeds at higher risk of MCT include the Boxer, Golden Retriever, Weimaraner, Labrador Retriever, Staffordshire Bull Terrier, Boston Terriers, Beagles, Schnauzers and the Pug (1, 5). MCTs are more common amongst older dogs, with dogs aged >10 years having 41 times higher odds of suffering from MCT than dogs aged <2 years (1).

Canine cutaneous MCT originate in the dermis and may extend into the subcutis. MCTs are heterogeneous in behavior and progression. Some are benign, developing slowly with minimal increase in size over time, while others progress rapidly to fatal metastatic disease (6). Current first-line therapy for localized, non-metastatic MCT is wide surgical resection (5–7). If complete margins are achieved and there is no evidence of metastasis, surgery is often curative. However, recurrence is common. For example, up to 27% of dogs with grade 2 MCT will have recurrence following surgery (6). When surgery is not possible, one or a combination of chemotherapy, radiation therapy or cytoreductive surgery is undertaken (5). These therapeutic options are often costly and not readily available to dogs in remote regions. The demand for effective alternative or adjunctive therapy to surgery for MCT is high.

Tigilanol tiglate (EBC-46) is a novel diterpene ester extracted from the seed of *Fontainea picrosperma*, a plant unique to Australian native rainforests. Discovered by the QBiotics Group, tigilanol tiglate represents a new class of drug that destroys tumors by modifying cell signaling processes and inducing rapid hemorrhagic necrosis of the treated tumor. Tigilanol tiglate is a potent activator of protein kinase C (8), a family of enzymes that modulate diverse cellular responses (9, 10). Intratumoral (IT) administration of tigilanol tiglate induces mitochondrial swelling and plasma membrane destruction in tumor cells (8). However, IT treatment with the drug primarily targets tumor vasculature increasing tumor vascular endothelial permeability resulting in PKC-dependent tumor vasculature hemorrhagic necrosis and rapid tumor ablation (8).

Preclinical studies in mice indicate that IT injection results in low levels of tigilanol tiglate in systemic circulation relative to subcutaneous injection into normal skin.

The antineoplastic potential of tigilanol tiglate has been demonstrated in mouse and rat studies as well as case studies treating dogs with cutaneous tumors including MCT and other round cell tumors, skin and subcutis soft tissue sarcomas, squamous cell carcinomas and oral melanomas (8, 11). A potential efficacious dose of 1.0 mg/mL tigilanol tiglate at 50% v/v tumor was determined from exploratory MCT case studies and was used as the starting dose for this study. Treatment of MCT with tigilanol tiglate usually does not require anesthesia or sedation, which is an important consideration given the age and often compromised nature of many dogs presenting with MCTs. Following destruction of the MCT with tigilanol tiglate, the deficit or “wound” remaining generally heals rapidly without the need for intervention such as oral or topical antibiotics or complex dressings. Healing is characterized by rapid granulation tissue development and subsequent full re-epithelialization of the wound. Healing generally occurs within 1 month (12).

The primary objective of this study was to characterize a safe and effective IT dose of tigilanol tiglate for the treatment of canine cutaneous MCT. The secondary objective of the study was to investigate the systemic concentrations of tigilanol tiglate following IT injection. This study was undertaken to select an appropriate dose of tigilanol tiglate for investigating the drug's efficacy and safety in pivotal clinical trials for the treatment of MCTs in dogs.

## Materials and Methods

This was a multicenter, open label, uncontrolled, non-randomized dosage characterization study using dose de-escalation. Dogs that satisfied the eligibility criteria were enrolled in the study as they presented to participating veterinary practices. The study was constructed as 4 descending dose cohorts being 1.0, 0.5, 0.2, and 0.05 mg/mL (cohorts 1–4, respectively) and delivered by IT injection as 50% v/v tumor. Patient recruitment was intended to be 10 dogs per cohort. The highest commencement dose (1.0 mg/mL) was selected as an intention-to-treat dose determined by case study investigations. Each cohort was fully recruited before the next, descending, cohort commenced recruitment.

### Patients

All dogs in the study were client owned animals. Patients were required to meet all of the study inclusion and exclusion criteria (listed below) and to have owner informed consent prior to enrolment. Each dog was then examined for overall health and wellbeing and the patient's tumor measured (length, width, and breadth) and tumor volume calculated (13) to determine whether it satisfied the study's tumor volume inclusion criterion (0.1–6.0 cm^3^). A fine needle aspirate was then taken to confirm diagnosis of cutaneous MCT. This use of a fine needle aspirate for diagnosis of MCT was adopted for three reasons: (1) the drug is administered by IT injection and taking a biopsy would have potentially confounded the study results due to possible leakage of some injected product from the biopsy site, (2) tumor grade was not a specific study criterion and (3) to minimize the risk of MCT degranulation. Additional inclusion criteria in this study were MCT stage I or IIa as defined by the WHO staging system for MCT (see Table 1) (14); MCT of first presentation in the dog, or MCT that appears at a distinct site to a previous MCT that was treated by surgical resection > 6 months previously; dog life expectancy > 12 months; weight of dog ≥ 5 kg; adequate hepatic, renal, and hematological function as determined by clinical assessment; and no evidence of gastrointestinal bleeding or coagulopathy. Dogs were excluded from the study if they had a MCT stage IIb, III, or IV; a subcutaneous MCT; prior radiation therapy or systemic chemotherapy for the treatment of the MCT; evidence of serious systemic MCT disease; pregnant, lactating or intended for breeding purposes; or had been administered corticosteroids within 14 days prior to enrolment in the study.

**Table 1:** WHO clinical staging system for mast cell tumors (14).

**Stage**	**Description**	
0	One tumor incompletely excised from the dermis without regional lymph node involvement	a. Without systemic signs b. With systemic signs
I	One tumor confined to dermis without regional lymph node involvement	a. Without systemic signs b. With systemic signs
II	One tumor with regional lymph node involvement	a. Without systemic signs b. With systemic signs
III	Multiple dermal tumors or large infiltrating tumor with or without regional lymph node involvement	a. Without systemic signs b. With systemic signs
IV	Any tumor with distant metastasis or recurrence with metastasis (including blood or bone marrow involvement)	

A dog could be removed prior to study completion if at any time, in the opinion of the investigator, the dog's welfare was at risk due to non-response of the tumor to tigilanol tiglate or due to a serious adverse event. An enrolled dog could also be removed from the study if the owner elected to voluntarily withdraw the dog from study; it was determined during the study that the dog did not meet the eligibility criteria; the dog required a medication prohibited by the protocol; the dog was deemed unsuitable for continuation in the study independent of health issues (e.g., the dog was fractious or uncooperative; owner non-compliance, etc.).

### Study Design

At screening, dogs were assessed for suitability for entry in the study. This included confirmation of cutaneous MCT via fine needle aspirate, clinical examination and tumor assessment. On pre-treatment day (day−1), mandatory prophylactic medication to prevent paraneoplastic MCT-induced adverse events was administered (**Table 3**). On treatment day (day 0), the target tumor volume was calculated and the designated dose of tigilanol tiglate administered via IT injection. Blood samples were collected at 0.5, 1, 2, 4, 8, and 24 h post-treatment to determine tigilanol tiglate plasma concentrations (pharmacokinetic analysis). Mandatory prophylactic medication was administered as per the protocol. Post-treatment clinical examinations and tumor assessments were performed on days 1, 2, 7, 14, and 21. Routine hematology and serum biochemistry samples were collected pre-treatment (day 0) and at days 7 and 21 post-treatment. The schedule of study activities is summarized in Table 2. All dogs were hospitalized overnight (day 0) and were discharged on day 1 if no adverse events were apparent. Dogs experiencing adverse events remained hospitalized until the adverse event resolved.

**Table 2:** Schedule of study activities.

**Activity**	**Screening**	**Pre-Tx** **−1d**	**Tx** **0d**	**Post-Tx**									
				**0.5 h**	**1 h**	**2 h**	**4 h**	**8 h**	**24 h**	**1–5d**	**7d** **± 1d**	**14d** **± 1d**	**21d** **± 2d**
Owner consent	X												
FNA	X												
Prophylactic medication		X	X							X			
Tigilanol tiglate injection			X										
Clinical examination & body weight	X	X							X		X	X	X
Tumor measurements	X	X	X								X	X	X
Hematology, clinical biochemistry		X									X		X
Plasma profiling		X		X	X	X	X	X	X				

*FNA, Fine needle aspiration; Tx, treatment; d, day; h, hour*.

### Tigilanol Tiglate Treatment

Tigilanol tiglate dosing was determined according to tumor size, and was delivered intratumorally at 0.5 mL per cm^3^ of tumor volume (50% v/v tumor). For robust in-clinic use, digital calipers were provided for measuring tumor length, width, and depth (13). MCT volume was then determined using a modified ellipsoid method ½ (length [cm] × width [cm] × depth [cm]), suggested as one of the most accurate volume calculations for a palpable tumor (15, 16).

Where possible, the tumor, and a 2 cm border surrounding the tumor, was shaved prior to treatment. This allowed for better visualization and recording of subtle skin changes including bruising and swelling of the skin surrounding the tumor site and easier management of the tumor site following necrosis. In some cases, this was not performed when it would cause undue stress to the animal or manipulation of the tumor. Study dogs were treated according to their cohort (allotted) tigilanol tiglate dose concentration. The required volume of the designated dose concentration of tigilanol tiglate was drawn into an appropriate 1 or 3 mL luer lock syringe fitted with a 23 gauge needle. The needle was then inserted into the tumor at a single injection point and moved in a radial manner in 2- and 3-dimensions with ~0.1 mL of drug delivered to each 0.2 cm^3^ of the tumor mass. No sedation or anesthesia was mandated.

Selected prophylactic and supportive medication were deemed mandatory for use in this study to prevent paraneoplastic MCT-induced adverse events such as Darier's sign and gastric upset. This medication included cetrizine, chlorpheniramine, omeprazole, acetylpromazine, methadone, and meloxicam (refer to Table 3 for details). To manage the anticipated localized transient pathology (bruising, swelling, and pain) associated with the mode of action of the drug and local inflammatory response at the injection site and subsequent inflammation, analgesia (either fentanyl patch or tramadol) was available for use if required. In addition, the antibiotic, amoxicillin/clavulanic acid, could be used supportively if required.

**Table 3:** Mandatory prophylactic and supportive medications.

**Medication (dose)**	**Class**	**Pre-Tx**		**Post-Tx**					
		**Day−1**	**Day 0**	**Day 0**	**Day 1**	**Day 2**	**Day 3**	**Day 4**	**Day 5**
Cetrizine (oral) 0.25 mg/kg BWt SID	H_1_ antihistamine	X		X	X	X	X	X	X
Chlorpheniramine (IM)	H_1_ antihistamine		X						
5 mg for ≤ 10 kg BWt									
5 mg per 10 kg (> 10 kg BWt)									
Omeprazole (oral)	Proton pump inhibitor	X		X	X	X	X	X	X
1.0 mg/kg BWt SID									
Acetylpromazine (SC)	Sedative & antiemetic		X						
0.005–0.2 mg/kg BWt									
Methadone (SC)	Analgesic		X						
0.04 mg/kg BWt									
Meloxicam (SC)	Anti-inflammatory & analgesic		X						
0.2 mg/kg BWt									
Meloxicam (oral)	Anti-inflammatory & analgesic				X	X	X	X	X
0.1 mg/kg BWt SID									

*Tx, treatment; IM, intramuscular; SC, subcutaneous; BWt, bodyweight; SID, once daily dosing*.

### Outcome Measures

Injection site reactions and tumor dimensions were assessed according to the schedule of activity (see Table 2). Determination of efficacy was based on objective tumor measurements made according to the Response Evaluation Criteria in Solid Tumors (RECIST) v.1.1 guideline (17) using the longest unidirectional tumor measurement (diameter). RECIST criteria were only applied to target tumors as tigilanol tiglate is not a systemic therapy. Response to therapy was defined as complete response (CR) resolution of the target tumor, partial response (PR) at least 30% decrease in the longest diameter of target tumor, stable disease (SD) decrease in the longest diameter of the target tumor of <30% or an increase of <20%, or progressive disease (PD) >20% increase in the longest diameter of the target tumor.

Clinical examinations were performed on days 1, 7, 14, and 21 post-treatment and dogs were evaluated for adverse events. Adverse events were defined as any unfavorable and unintended sign, symptom, or disease temporally associated with the use of tigilanol tiglate, whether or not related to the medication. The severity of adverse events were graded according to the veterinary cooperative oncology group—common terminology criteria for adverse events (VCOG-CTCAE) following chemotherapy or biological antineoplastic therapy in dogs and cats grading system (18).

Plasma profiling of tigilanol tiglate was performed over the first 24 h following IT administration. For each dog, plasma concentrations were used to determine the maximum plasma concentration (C_max_), time to maximum concentration (T_max_), area under the curve to the last time point (AUC_last_) and extrapolated to infinity (AUC_inf_), the terminal elimination rate (λ_z_) and the terminal half-life (t_½_).

Body weight and body weight changes during the study were compared between the three cohorts to determine any treatment effects. Serum biochemistry included the analysis of creatinine, protein, albumin, globulin, alkaline phosphatase, ALT, AST, gamma GT, creatine kinase, cholesterol, triglycerides, magnesium, calcium, phosphate, sodium, potassium, chloride, glucose, urea, amylase, and bilirubin. Hematology included analysis of red blood cell count, white cell count, white cell differential count, platelets, packed cell volume, mean corpuscular volume, mean corpuscular hemoglobin, mean corpuscular hemoglobin concentration, hemoglobin, reticulocytes, prothrombin time, and activated partial thromboplastin time.

### Statistical Analysis

Pairwise comparison of categorical responses to treatment between treatment groups at each time point post-treatment were compared using Fisher's Exact Test. Using the RECIST classification (17), tumor data was analyzed to determine the effectiveness of, and differences between, treatments. Generalized linear modeling was used to examine the combined effects of treatment dose, time post-treatment and other potentially exploratory variables. Generalized estimating equations with appropriate error structure (and controlled for repeated measurements of individuals) were used to examine categorical response to treatment and changes in tumor volume measurement. Tumor volume analysis was also controlled for pre-treatment tumor volume.

### Statement on Welfare of Animals

Study dogs were managed similarly and with due regard for their welfare. Dogs were handled in compliance with University of New England (Armidale NSW, Australia) animal ethics authority. The protocol was reviewed and approved by the aforementioned animal ethics authority, approval number AEC No. 12–121. Study dogs were housed individually in cages within the veterinary practice for the first 24–48 h of the study in accordance with protocol and at the investigator's discretion. After this, dogs were released to owner care (if their condition was stable) and they remained with their owner following completion of the study.

## Results

Thirty dogs were recruited into the study of which there were 27 evaluable dogs. Three dogs, all from cohort 3 (0.2 mg/mL), were excluded from the analysis as two were under-dosed due to calculation errors, with the third excluded as a significant quantity of tigilanol tiglate failed to be injected into the tumor. The study was terminated prior to recruiting dogs of cohort 4 (0.05 mg/mL) due to the lower response rate observed in cohort 3 (0.2 mg/mL).

Amongst the evaluable dogs, 20 were female and 7 were male. The median age at study enrolment was 8.0 years (range 2–15 years) and median bodyweight was 24.0 kg (range 7.4–38.9 kg). The most common breeds represented were Staffordshire Terrier or cross (*n* = 9), and Labrador Retriever or cross (*n* = 4). The median tumor volume on the day of treatment was 0.66 cm^3^ (range 0.16–4.69 cm^3^). At a dose level of 0.5 mL tigilanol tiglate per cm^3^ tumor, the median dose volume was 0.35 mL (range 0.08–2.35 mL) (see Table 4).

**Table 4:** Patient demographics and treatment details.

**Cohort**	**Breed**	**Age (years)**	**Sex**	**Tumor location**	**Tumor volume (cm^**3**^)**	**Tigilanol tiglate conc. (mg/mL)**	**Dose volume (mL)**	**Tigilanol tiglate dose (mg)**
1	Labrador Retriever X	8	M	Left forelimb	2.90	1.0	1.45	1.45
1	Staffordshire Terrier	12	F	Right base of tail	0.65	1.0	0.35	0.35
1	Labrador Retriever	5	F	Left mammary	1.62	1.0	0.80	0.80
1	Rhodesian Ridgeback X	8	F	Left base of tail	0.42	1.0	0.20	0.20
1	Boxer	7	F	Left thoracic wall	0.90	1.0	0.45	0.45
1	Australian Border Collie	8	F	Right forefoot	0.81	1.0	0.40	0.40
1	Pug	6	F	Right hindlimb	1.69	1.0	0.85	0.85
1	Staffordshire Terrier	6	F	Left rump	0.73	1.0	0.36	0.36
1	Labrador Retriever	8	F	Left forelimb	1.73	1.0	0.85	0.85
1	Staffordshire Terrier	8	M	Left perianal	4.69	1.0	2.35	2.35
2	Staffordshire Terrier X	13	F	Left hindlimb	1.87	0.5	0.95	0.48
2	Mastiff	2	F	Vulva	0.36	0.5	0.20	0.10
2	Maltese X	7	F	Right maxilla	4.03	0.5	2.00	1.00
2	Siberian Husky X	7	F	Right hindlimb	0.36	0.5	0.20	0.10
2	Staffordshire Terrier	6	F	Right flank	0.17	0.5	0.10	0.05
2	Bull Mastiff X	10	M	Right pelvis	1.20	0.5	0.60	0.30
2	Neopolitean Mastiff X	6	M	Right thoracic wall	0.77	0.5	0.38	0.19
2	Staffordshire Terrier	8	M	Right shoulder	0.26	0.5	0.13	0.07
2	Basenji	15	F	Left side axilla	4.15	0.5	2.05	1.03
2	Staffordshire Terrier X	8	M	Periocular	0.36	0.5	0.20	0.10
3	Staffordshire Terrier	7	F	Left thoracic wall	0.39	0.2	0.20	0.04
3	Staffordshire Terrier	8	F	Left mammary	0.16	0.2	0.08	0.02
3	Maltese	12	M	Right flank	0.51	0.2	0.30	0.06
3	Boxer	4	F	Right hindlimb	0.66	0.2	0.35	0.07
3	Labrador Retriever	10	F	Base of right ear	0.43	0.2	0.22	0.04
3	Pharaoh Hound	8	F	Vulva	0.30	0.2	0.15	0.03
3	Terrier	5	F	Left lateral thigh	0.22	0.2	0.11	0.02

*conc., concentration*.

Patients enrolled in cohort 3 (0.2 mg/mL) had significantly smaller tumors than those enrolled in cohort 1 (1 mg/mL) as assessed by tumor longest axis length at pre-screening (*P* < 0.05) and tumor maximum diameter on the day of treatment (*P* < 0.05). Tumor sizes were not significantly different between cohorts 2 and 3 nor 1 and 2.

### Response to Treatment

**Figure 1:**
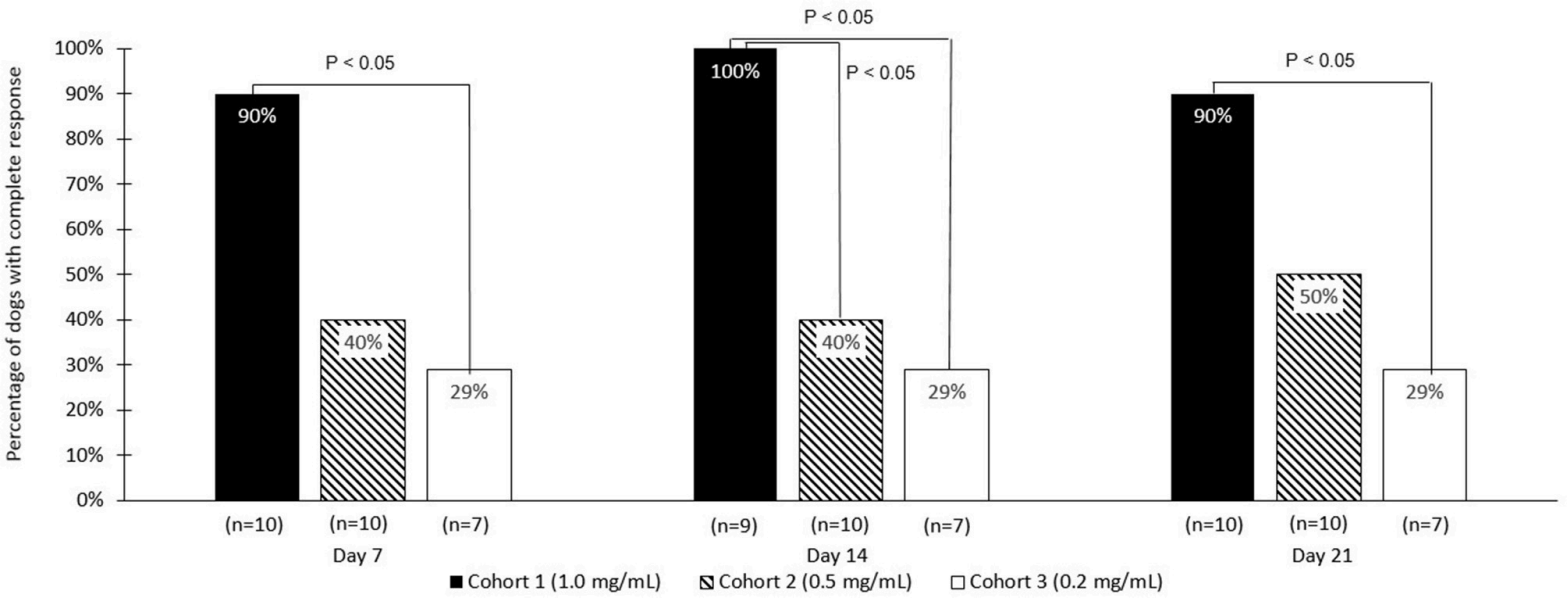
Comparative complete response rate (based on RECIST criteria) for the three tigilanol tiglate dose cohorts at days 7, 14, and 21.

**Figure 2:**
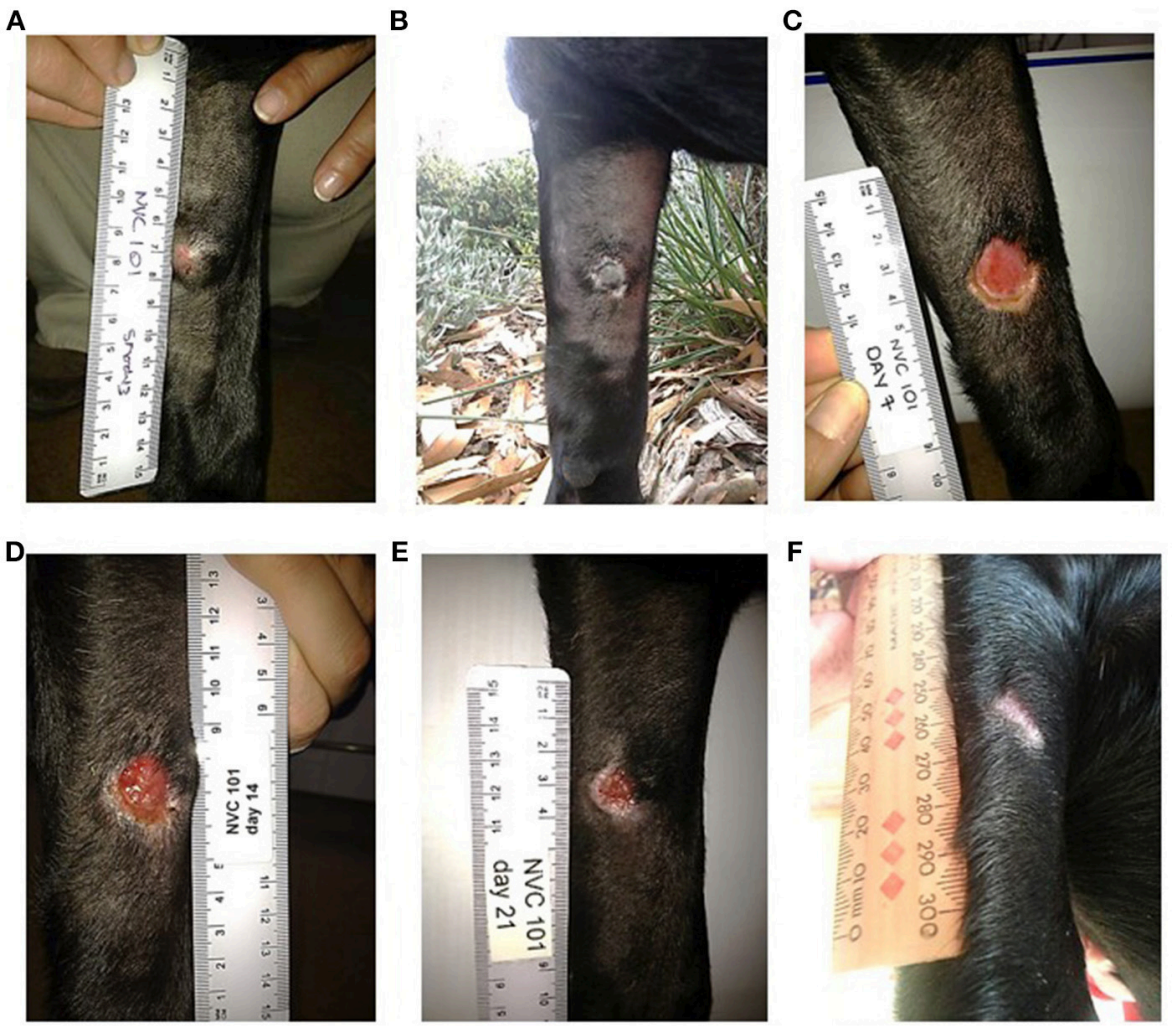
Complete response following intratumoural injection of tigilanol tiglate. **(A)** MCT prior to treatment. **(B)** 24 hours post treatment. **(C)** 7 days post treatment. **(D)** 14 days post treatment. **(E)** 21 days post treatment. **(F)** 37 days post treatment.

The strongest response to treatment, as assessed by the proportion of dogs achieving RECIST classification of CR, was observed amongst dogs who received the highest dose of tigilanol tiglate (cohort 1, 1 mg/mL) with a CR, *P* < 0.05 (see Figure 1 and Table 5). Amongst these dogs, 9 experienced CR and 1 experienced SD. Note, the dog in cohort 1 that experienced SD was not assessed on day 14. Of the 10 dogs treated in cohort 2 (0.5 mg /mL), 5 experienced CR, 1 PR, 3 SD, and 1 PD. Of the 7 dogs treated in cohort 3 (0.2 mg/mL), 2 experienced CR, 1 PR, and 4 SD. Response to tigilanol tiglate therapy was rapid, with all but one of the CRs occurring within 7 days of treatment. Typical response to tigilanol tiglate, tumor necrosis and subsequent healing, is illustrated in the photographs in Figure 2.

**Table 5:** RECIST response rate at days 7, 14, and 21.

**Tigilanol tiglate dose**	**Day 7**		**Day 14**		**Day 21**	
	**RECIST non-CR**	**RECIST CR**	**RECIST non-CR**	**RECIST CR**	**RECIST non-CR**	**RECIST CR**
0.2 mg/mL (*n* = 7)	5	2	5	2	5	2
0.5 mg/mL (*n* = 10)	6	4	6	4	5	5
mg/mL (*n* = 10)	1	9	0^*^	9	1	9
Statistical difference	1 mg/mL significantly better than 0.2 mg/mL (*P* < 0.05)		1 mg/mL significantly better than 0.2 mg/mL and 0.5 mg/mL (*P* < 0.05)		1 mg/mL significantly better than 0.2 mg/mL (*P* < 0.05)	

*CR, complete response; Non-CR, patients with either partial response, stable disease, or progressive disease*.

* *One dog in cohort 1 was not assessed on day 14*.

### Adverse Events

The 27 dogs treated on this trial experienced a total of 64 adverse events (see Table 6). The majority (81.3%) of adverse events were mild (VCOG-CTCAE classification grade 1) with no intervention required. The remaining adverse events were all classified as grade 2, requiring minimal medical intervention such as the use of oral antibiotics or additional analgesics. All adverse events were transient in nature. Half of the reported events related to the anticipated local pathology generated by tumor necrotic action of tigilanol tiglate on the MCT which produces transient localized pain and swelling. Dogs from cohort 1 who received the highest dose of tigilanol tiglate experienced the lowest number adverse events with a mean of 1.8/dog, compared to 3.1/dog in cohort 2 and 2.1/dog in cohort 3. Dogs in cohort 1 experienced only two grade 2 adverse events, being one case of wound infection and one case of otitis external requiring antibiotic therapy.

**Table 6:** Incidence and VCOG-CTCAE grading of adverse events.

**Adverse event (medical intervention)**	**All patients (*****n*** **=** **27)**		
	**No of events**	**Incidence (%)**	**VCOG-CTCAE grade^†^**
Local swelling at treatment site^*^	22	81.5	1
Pain associated with treatment site^*^	4	14.8	1
Pain associated with treatment site^*^ (oral analgesic)	6	22.2	2
Transient tachypnoea	14	51.9	1
Transient lethargy	5	18.5	1
Transient tachycardia	2	7.4	1
Bilateral otitis external (oral antibiotic)	1	3.7	2
Transient elevated blood pressure reading	1	3.7	1
Transient salivation	1	3.7	1
Emesis	2	7.4	1
Flatulence (oral antibiotic)	1	3.7	2
Seborrhoea (oral antibiotic)	1	3.7	2
Corneal ulcer (antibiotic ointment and oral analgesic)	1	3.7	2
Wound sinus	1	3.7	1
Wound infection (oral antibiotic)	2	7.4	2
Total number of adverse events	64		

* *Expected event due to the mode of action of Tigilanol tiglate*.

† *Grade 1 (Mild); asymptomatic or mild symptoms; clinical signs or diagnostic observations only; intervention not indicated. Grade 2 (Moderate): minimal, out-patient or non-invasive intervention indicated; moderate limitation of activities of daily living*.

Serum biochemistry findings were generally unremarkable with the exception of 2 dogs; one dog (cohort 2) with slightly elevated aspartate aminotransferase (AST) and elevated creatine kinase readings post-treatment, and the second dog (cohort 3) with transiently elevated AST and alanine aminotransferase (ALT) readings post-treatment. Both dogs appeared normal and in good health throughout the study.

Hematology observations were generally unremarkable with some exceptions, all of which were considered not clinically meaningful. Seven dogs (3 dogs in cohort 1, 3 dogs in cohort 2 and 1 dog in cohort 3) had elevated reticulocytes readings, but none were associated with abnormal packed cell volume measures or anemia. Five dogs (2 dogs in cohort 1 and 3 dogs in cohort 2) had slightly reduced platelet counts. However, in all cases the platelets were described as “clumping” which can falsely lead to lower counts on automated analyzers (19).

### Body Weights

The mean change in body weight across all dogs over the course of the study was −0.6% (σ: 4.4%). Dogs in cohort 1, who experienced the overall best response rate, on average gained 1.7% (σ: 5.1%) in body weight, with 6 dogs gaining weight and 3 dogs losing weight. Nine of the dogs in cohort 2 lost body weight, with the mean change in body weight being −2.6% (σ: 1.8%). In cohort 3, 3 dogs gained weight and 4 dogs lost weight with the mean change being −1.1% (σ: 5.0%).

### Plasma Profile Analysis

**Figure 3:**
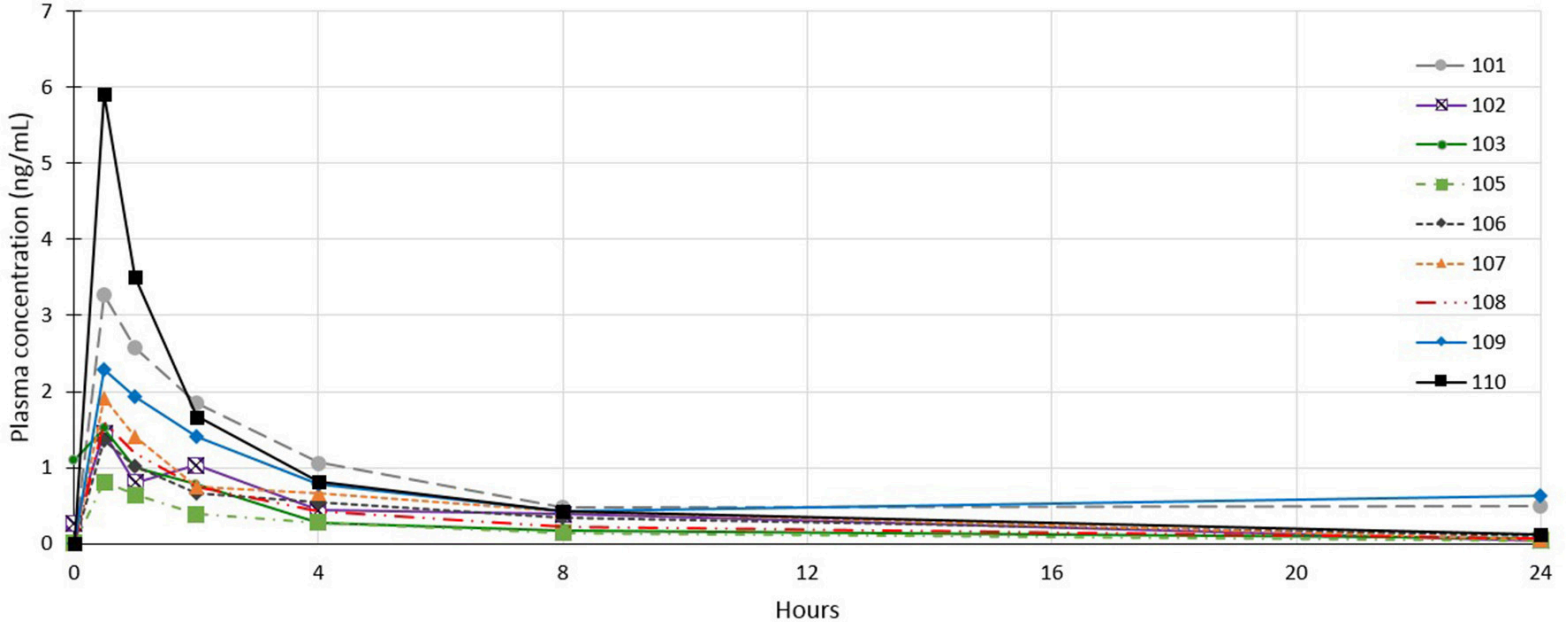
Individual plasma concentration curves for dogs in cohort 1 who received tigilanol tiglate (1 mg/mL).

Plasma profile analysis was evaluable in 26 dogs, with one dog from cohort 1 excluded as no samples were collected due to aggressive in-clinic behavior. Plasma concentration curves were typical of a non-intravenous parenteral administered medication (see Figure 3). C_max_ and T_max_ occurred at the first time point (30 min) for all but 5 dogs (1 dog in cohort 2; T_max_ = 1 h, C_max_ = 0.08 ng/mL and 4 dogs in cohort 3; T_max_ = 1 h for 2 dogs (C_max_ values of 0.36 and 0.54 ng/mL) and 2 h for 2 dogs [C_max_ values of 0.05 and 0.40 ng/mL)]. The mean T_max_ was 0.67 h (σ: 0.42 h). In most dogs, plasma concentrations of tigilanol tiglate were below measurable at 24 h after dosing (see Figure 3). The mean plasma half-life of tigilanol tiglate was 6.53 h (σ: 3.03 h).

**Figure 4:**
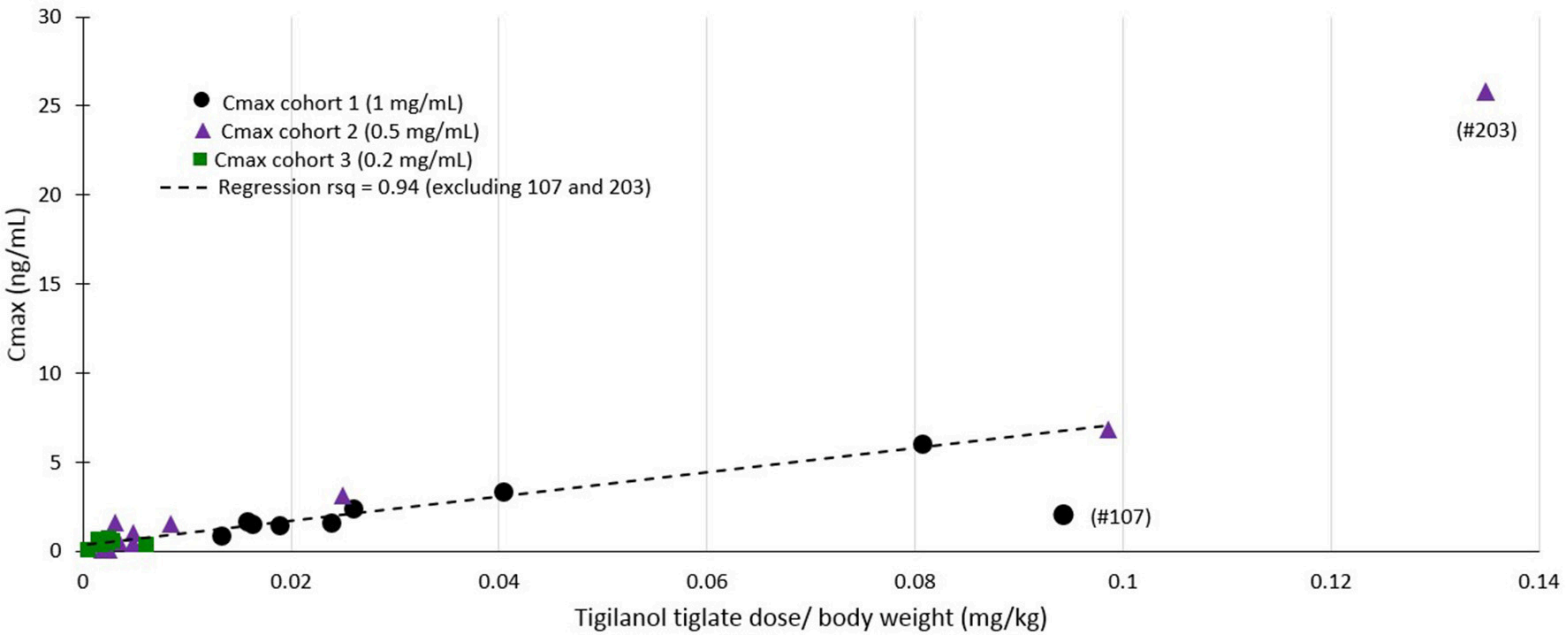
Cmax vs. dose normalized by body weight.

Regression modeling demonstrated a consistent relationship between the amount of tigilanol tiglate dosed per body weight and C_max_ (*r*^2^ = 0.94) (see Figure 4) as well as systemic exposure AUC_last_ (*r*^2^ = 0.59), excluding two outliers (dogs 107 and 203).

## Discussion

Dogs treated with the highest dose of tigilanol tiglate (1 mg/mL) experienced the highest response rate, with 90% of dogs attaining a CR according to RECIST classification. This response rate was statistically superior to the lowest dose, 0.2 mg/mL, at all time points and superior to 0.5 mg/mL dose at day 14 (*P* < 0.05), (however note the missed data for 1 dog). The highest dose of tigilanol tiglate was also associated with the lowest frequency of adverse events per dog with the vast majority of adverse events being mild and transient. Based on the overall clinical response from this study, a suitable dose of tigilanol tiglate for the treatment of MCT in dogs appears to be 1 mg/mL administered IT at 0.5 mL per cm^3^ of tumor volume (50% v/v tumor).

Analysis of tumor size identified a potential bias in the study, with dogs entered in cohort 3 having significantly smaller tumors than those in cohort 1. As larger MCTs are likely to be more advanced and more progressive than smaller tumors, it is reassuring that the highest response rate was observed in cohort 1 which had the largest tumors. Thus, this potential recruitment bias further validates the superior efficacy of tigilanol tiglate dosed at 1 mg/mL.

Clinical examinations, serum biochemistry and hematology investigations were clinically unremarkable and reinforced the good safety profile of IT tigilanol tiglate at all three dose levels. Bodyweights did exhibit some fluctuations with both gains and losses experienced on study. However, these body weight fluctuations were not extreme and not dose-related. Clinical attendance and increased handling of certain dogs may have contributed to weight loss, although these weight changes may be unrelated to the study such as change in feeding regimes. On the few occasions when serum biochemistry or hematology results were outside normal values, the distribution of these events across the three cohorts does not suggest any dose-relationship.

Plasma profiling of tigilanol tiglate was typical of a non-intravenous parenteral administered medication. Peak concentrations occurred soon after administration and was followed by rapid systemic clearance. As observed in preclinical studies (8) systemic exposure to tigilanol tiglate is proportionate to the delivered dose and is short lived with most patients having no detectable tigilanol tiglate in plasma within 24 h of IT injection.

The high efficacy rate and good tolerability reported here, combined with no requirements for anesthetic or sedation in most cases, supports the potential of tigilanol tiglate as an alternative to surgery for the treatment of MCT in dogs. In addition, the drug would appear to have some advantages over many current chemotherapy treatments for MCT and other solid tumors in that it is delivered by direct injection into the tumor mass which (a) immediately locates the drug to its site of action and (b) minimizes possible side-effects that are often associated with systemic delivery (i.e., intravenous, intramuscular, or oral) of drugs where non-target tissues and organs are also often adversely affected (20, 21).

## Concluding Remarks

This dose characterization study identified that tigilanol tiglate at a concentration of 1 mg/mL dosed at 0.5 mL per cm^3^ of tumor volume (50% v/v tumor), is highly efficacious for the treatment of MCT in dogs. The drug was also demonstrated to be well-tolerated and safe for the patient population treated, with adverse events being mild and of short duration. These results support further development of tigilanol tiglate for the treatment of MCT, and potentially other solid tumors, in dogs and underpins dosing regimen for future studies.

Based on this study, a fully blinded and controlled pivotal efficacy study with tigilanol tiglate treating MCT in dogs has been undertaken in the USA (results to be reported) and a similar efficacy study treating soft tissue sarcomas in dogs is currently underway in the UK and France.

## Ethics Statement

Study dogs were managed similarly and with due regard for their welfare. Dogs were handled in compliance with University of New England (Armidale NSW, Australia) animal ethics authority. The protocol was reviewed and approved by the aforementioned animal ethics authority, approval number AEC No. 12–121. Study dogs were housed individually in cages within the veterinary practice for the first 24–48 h of the study in accordance with protocol and at the investigator's discretion. After this, dogs were released to owner care (if their condition was stable) and they remained with their owner following completion of the study.

## Author Contributions

JM and AB trial design, in-clinic conduct, data collection, manuscript writing contribution. VG, PR, PS, SL, and JC trial design, protocol writing, in-clinic trial monitoring and auditing, data verification, data collection, data analysis, manuscript writing contribution.

### Conflict of Interest Statement

VG, PS, PR, and SL are employed by QBiotics. At the time of the study being conducted JC was working at Tableland Veterinary Services as a practicing veterinarian. She now is an employee of QBiotics. QBiotics own the intellectual property and patents associated with Tigilanol tiglate. The remaining authors declare that the research was conducted in the absence of any commercial or financial relationships that could be construed as a potential conflict of interest.
